# The mediating role of exogenous shocks in green purchase intention: evidence from italian fashion industry in the Covid-19 era

**DOI:** 10.1007/s43039-023-00065-4

**Published:** 2023-01-19

**Authors:** Eleonora Annunziata, Tommaso Pucci, Jacopo Cammeo, Lorenzo Zanni, Marco Frey

**Affiliations:** 1 Institute of Management, Scuola Superiore Sant’Anna, Piazza Martiri della Libertà, 24, 56127 Pisa, Italy; 2 Department of Business and Law, University of Siena, Via Banchi di Sotto, 55, 53100 Siena, Italy

**Keywords:** Environmental knowledge, Environmental concern, Eco-friendly behaviour, Sustainable fashion, Purchase intention, COVID-19

## Abstract

This work investigates how the COVID-19 outbreak has affected consumers’ green purchasing intention in the fashion industry. It examines how this exogenous shock has influenced the relationship between consumer green purchasing intention and its three relevant antecedents (environmental knowledge (EK), environmental concern (EC), eco-friendly behaviour (EFB)). A survey of 1433 Italian consumers was carried out. The data were analysed through a structural equation modelling method to evaluate the direct and indirect mediating effects of the COVID-19 shock on the relationship between green purchasing intention and its antecedents (i.e., EK, EC and EFB) by considering traditional and online purchasing channels. The results of the model confirmed a positive relationship between green purchasing intention and its antecedents. The COVID-19 pandemic was also found to positively mediate the relationship between green purchase intention and two out of its three antecedents (i.e., EK and EC) in both traditional and online purchasing channels. This paper contributes to the literature on sustainable consumption by assessing magnitude of the relationship between green purchasing intention and its antecedents, and the mediating role exerted by a complex exogenous shock such as the COVID-19 outbreak.

**Supplementary Information:**

The online version contains supplementary material available at 10.1007/s43039-023-00065-4.

## Introduction

The environmental problems brought on by overproduction, such as global warming, air and water pollution, and difficult waste disposal, have become increasingly significant (United Nations, 2015). Companies have thus identified sustainability as a priority to react on associated societal pressure and/or increase economic success (Schaltegger and Horisch, 2017; Williams et al., 2017).

Consumers have started to avoid harmful products and have become more sensitive in terms of their environmental attitudes, preferences, and purchases (Sharma et al., 2020). In fact, a recent study carried out by the European Commission shows that European citizens consider that changing consumption patterns is the most effective way of tackling environmental problems. However, on average only 22% of European consumers recently bought products with an environmental label, with only 13% purchasing them in Italy. (Eurobarometer, 2020).

There is, therefore, a substantial amount of literature regarding the analysis of the so-called antecedents of green purchasing behaviour (Lee et al., 2014; Jung & Jin, 2014; Lin & Niu, 2018), and specifically regarding the beliefs that determine green purchase intention (Gam, 2011; Groening et al., 2018) highlighted that environmental knowledge, environmental concern, and eco-friendly behaviour are three relevant determinants of green purchasing intention still requiring further investigation. Previous studies on these three antecedents have provided conflicting results on their direct and indirect influence on green purchasing intention: environmental knowledge (Indriani et al., 2019), environmental concern (Onurlubaş, 2018) and eco-friendly behaviour (Blasi et al., 2020).

Scholars have identified the existence of contextual factors (Ertz et al., 2016) that can act as mediators in the abovementioned relationships, and which can be categorized as institutional support (Newton et al., 2015), cultural context (Onurlubaş, 2018), but also exogenous shocks (Tilikidou & Delistavrou, 2014).

The crisis resulting from the COVID-19 outbreak as an exogenous shock has led to epochal upheavals (Amed et al., 2021). The global economy has experienced the worst recession of the last century (Wildman, 2021), which has affected several industries (e.g., aviation, hospitality, tourism, entertainment, and transportation) (Rai et al., 2021) and particularly the fashion industry has experienced a worse economic performance than during the financial crisis in 2008 (Boston Consulting Group, 2020). Significant changes have triggered the implementation of more sustainable production and consumption practices due to the strong impact on people’s behavioural, normative and control beliefs determined by the pandemic (Foroudi et al., 2021).

This work builds on the existing literature on green purchasing, with a specific focus on the relationship between consumer purchasing intention and its three antecedents (environmental knowledge, environmental concern, eco-friendly behaviour), and assesses how COVID-19 challenged sustainable consumption in the fashion industry.

Gam (2011) argued that disentangling the determinants of the environmentally friendly purchasing of fashion products is more complex than other industries, since the fashion industry is one of “the most change-intense categories of consumer products” (Kunz et al., 2005) due to the social dynamics involved (e.g., fashion trends and seasonal changes). Moreover, fashion companies and their consumers are increasingly interested in tackling environmental issues (Amed et al., 2021). In fact, a recent survey of European Commission showed that on average 65% of European respondents were interested in knowing the environmental performance of their clothes (Eurobarometer, 2020).

Italy is a relevant research setting because the fashion industry is one of the most important sectors for Italian economy, accounting for around 1% of the National GDP and with a turnover of more 75 billion Euros (Confindustria Moda, 2021). In terms of quality, thanks to its well-known brands around the world (e.g., Armani, Gucci, Versace, etc.), and in terms of quantity, small and medium enterprises make up a large portion of the industry (Confindustria Moda, 2021). In addition, 56% of Italian citizens consider that information on the environmental performance of their apparel is important (Eurobarometer, 2020).

We identified three research gaps: conflicting results in the analysis of the magnitude of the relationship between green purchasing intention and its antecedents (Newton et al., 2015), a lack of studies on the mediating role of exogenous shocks on the abovementioned relationships (Ertz et al., 2016), and the lack of an in-depth assessment of the direct effect of the COVID-19 pandemic on green purchase intention.

Our study thus investigates how COVID-19 has impacted on the consumers’ green purchasing intention and how it mediates the relationship between the antecedents and this intention.

In Sect. 2, after a brief literature review, the hypotheses to be tested are presented. Considering the key role of e-commerce in the pandemic era (Laato et al., 2020), a distinction is highlighted in the conceptual model used in this section to show the mediating effect of the COVID-19 outbreak on purchasing channels: physical (traditional) and online.

Section 3 details the relevance of the empirical setting and the presentation of the sample and data collection, and measurement of the variables. Using a structural equation modelling method, the collected data are analysed, and the results of the measurement model are shown in Sect. 4. Section 5 discusses the key findings. Section 6 looks at managerial implications, limitations of the study and presents ideas for further research.

## Theoretical background and hypotheses

Green purchasing behaviour (GPB) belongs to the pro-environmental behaviour (PEB) construct because consumers buy environmentally friendly products that reduce the negative impact on the environment (Uddin and Kahn, 2018; Dagher et al., 2015).

The particular features of green purchasing as a subcategory of PEB have led to a growing literature on the analysis of its determinants (Lee et al., 2014; Joshi & Rahman, 2015; Ertz et al., 2016). Several studies based on rational-based theoretical models (i.e., the theory of reasoned action and the theory of planned behaviour) have revealed that intentions are a key determinant of behaviours (Chan & Lau, 2002; Ajzen, 2015) and thus purchasing behaviours (Moser, 2015; Lin & Niu, 2018). In addition, Park & Ha (2012) explained the relationships between the determinants of green purchasing, highlighting the predictive role of behaviour intention.

### Environmental knowledge, environmental concern, and eco-friendly behaviour as antecedents of green purchase intention

The main factors behind intentions are related to consumers’ social norms, values, and beliefs (Ertz et al., 2016), and attitudes are in turn affected by values and beliefs (Kumar et al., 2018), built from past experiences, current concerns, information, and social pressure (Gam, 2011). Within this framework, studies carried out on the relationship between green purchasing intention and its antecedents provide discordant results (Groening et al., 2018; Onurlubaş, 2018; Indriani et al., 2019; Blasi et al., 2020). Particularly, ambiguous results emerged from empirical research on the relationships between the antecedents and consumers’ environmental consumption behaviour in the fashion industry (Kim & Damhorst, 1998; Brosdhal and Carpenter, 2010; Khare 2019). For instance, Saricam and Okur (2019) demonstrated the relevance of attitude towards the behaviour as determinant of intention in their research on sustainable fashion, without analysing in detail the role of the antecedents.

Therefore, it is crucial to investigate the role played by knowledge and beliefs in green purchase intention.

Environmental knowledge regards the level of understanding of ecosystem dynamics and the consumers’ “awareness about the impact of manufacturing processes on the environment” (Khare, 2019). Previous studies have highlighted a potential correlation between environmental knowledge and green purchasing intention (Biswas & Roy, 2015; Indriani et al., 2019) or behaviour (Pickett-Baker & Ozaki, 2008).

In their model, Lin & Niu (2018) tested with good reliability that environmental knowledge has a significant effect on a person’s environmental attitudes, and that environmental attitudes, in turn, positively affect purchasing intention.

However, Vicente-Molina et al., (2013) showed that objective environmental knowledge may not necessarily foster green purchasing behaviour unless, as stated by Martin & Simintiras (1995), the knowledge regards the environmental characteristics of products and manufacturing processes.

Indriani et al., (2019) investigated the effects of environmental knowledge on green purchase intention (direct effect) but did not find a significant relationship. On the other hand, the relationship was significant and the analysis reliable in the case of the relation mediated by attitudes towards green products (indirect effect). Groening et al., (2018) argued that knowledge may not directly affect green product decision making. Sadiq et al., (2021) pointed out that the role of environmental knowledge in influencing green purchasing intention has been an unexplored area, especially in the context of green apparel. The positive link between knowledge and green purchasing intention in the fashion industry therefore needs further investigation since previous studies did not achieve significant results. Thus, the first hypothesis of the model is:

#### H1

Consumers with a greater environmental knowledge of production are more likely to have the intention to purchase sustainable fashion products.

Second, environmental concern can be defined as the general belief of some consumers (Fransson and Garling, 1999) for which environmental issues are important because they are worried about the threats to the environment (Lee et al., 2014; Amed et al., 2021).

Some studies have highlighted that consumers with high environmental concern are more likely to demonstrate green purchasing behaviours (Diamantopoulos et al., 2003). For example, Lee et al., (2014) confirmed that environmental concern has a positive significant influence on green purchasing behaviour. Theoretical models such as the theory of planned behaviour have shown that environmental concern is a predictor of green purchase intention (Gam, 2011; Jung & Jin, 2014).

Although environmental concern is considered as a direct antecedent of environmental purchase intention, there is no consensus regarding the magnitude of this relationship (Newton et al., 2015). Onurlubaş ( 2018) shows that environmental concern has a significant influence on purchase intention for green products. In their pivotal work on fashion consumption, Kim & Damhorst (1998) showed that environmental concern of clothing consumers did not clearly relate to environmentally responsible apparel consumption. Hartmann & Apaolaza-Ibáñez (2012) studied how environmental concern positively affects the intention to purchase green-branded energy, finding a highly significant relationship between those variables. Therefore, the second hypothesis tested in this study is:

#### H2

Consumers with a greater environmental concern are more likely to have the intention to purchase sustainable fashion products.

Despite the heterogeneous structure of PEB (Stern, 2000; Turaga et al., 2010), most studies have emphasized the private sphere behaviours of PEB that are every day and widespread actions with several positive environmental impacts such as recycling, waste reduction, etc. (Larson et al., 2015). Therefore, in the individual private sphere, eco-friendly behaviours might represent a predictor of future behaviour since they show consumers’ ability to engage in other sustainable behaviours (Phau et al., 2014; Kim et al., 2016; Magnuson et al., 2017). However, previous studies have detected both direct and indirect effects (Abdul-Muhmin, 2007). More recent studies have found a positive relationship between eco-friendly behaviour and green consumption in the fashion industry (Brosdhal and Carpenter, 2010; Park & Oh 2014), but Blasi et al., (2020) referred to this relationship as “scattered and mostly anecdotal” and requiring further investigation to achieve more robust results. Thus, the third hypothesis tested in this study is:

#### H3

Consumers with eco-friendly behaviour are more likely to have the intention to purchase sustainable fashion products.

### The effect of the COVID-19 outbreak on the determinants of green purchase intention

Contextual factors, together with attitudes and norms, are important determinants of green purchasing intention and related behaviours (He & Harris, 2020). An upturn of contextual factors can arise from an exogenous shock (e.g., economic crisis, natural disaster, etc.) (Ozdamar Ertekin et al., 2020). The COVID-19 outbreak is one such case. The pandemic led to a series of upheavals affecting many economic sectors (Donthu & Gustafsson, 2020), challenging social practices (Van Bavel et al., 2020), negatively impacting human life (Cruz-Cárdenas et al., 2021), and testing the resilience of organisations (Rai et al., 2021).

In fact, the COVID-19 shock was not just an economic crisis (Pantano et al., 2020) but a systemic and disruptive crisis (Eger et al., 2021). The decrease in human activity during the pandemic’s most crucial phase had a favourable impact on the environment, which indirectly aided in the partial restoration of natural ecosystems (Bar, 2021). However, the consequences of the COVID-19 pandemic from an environmental point of view are thus very complex (Brydges et al., 2020).

The impact of the COVID-19 outbreak has quickly taken a key place in the academic debate. However, green purchasing is not yet among the main themes investigated (Verma & Gustafsson, 2020). To date, the literature has only provided a few assumptions: this pandemic may have boosted sustainability issues within companies and consumers (Hasbullah et al., 2020), or, in contrast, may have triggered controversial mechanisms and habits (e.g., the emissions generated by express deliveries) (Xie et al., 2020).

The impact of an exogenous shock on the dynamics of green purchasing may not be unique. Tilikidou & Delistavrou (2014) demonstrated the controversial effects of the Greek financial crisis of the early 2010s on green purchasing where a reduction in consumption was not accompanied by higher green purchases. The COVID-19 pandemic also disrupted consumption habits globally (Cambefort, 2020), as happened after the financial crisis in 2008 when people started to understand the impact of their consumption on the general society and the environment (Bondy & Talwar, 2011).

These changes took place in a climate of uncertainty and fear that influenced consumer behaviour (Ioannides and Gyimothy, 2020): the reduction in consumers’ purchasing capacity is one of the clearest effects of the economic crises (Ozdamar Ertekin et al., 2020). According to Mehta et al., (2020) the social isolation and the less availability of resources resulting from the pandemic could lead consumers to reduce their consumption. The COVID-19 pandemic also gave consumers time to look for alternatives, consider their long-term ethical consumer choices, and change their consumption behaviour though a greater environmental awareness (Sheth, 2020).

Pantano et al., (2020) highlighted the need to investigate how the emergency of the pandemic could push towards more sustainable purchasing behaviours. Previous research has shown that the pandemic has accelerated the consumers’ trend towards more sustainable consumption in specific traditional sectors like food and transports (Brzustewicz and Singh, 2021). Orîndaru et al., (2021) noticed that the purchase decisions in physical stores became more prudent, with a growing interested in buying local, fresh or zero miles products instead of processed or semi-processed ones. Chae (2021), on the contrary, suggested that the pandemic could have increased people self-centeredness and consumers’ perceived threat, leading them to neglect green purchasing, when they went to physical stores, in favour of cheaper, less sustainable products. Moreover, Brydges et al., 2020 argued that the relationship between COVID-19 and green consumption in the fashion industry has been not yet clearly verified. Therefore, an investigation of the positive effect of the COVID-19 on the green purchase intention in traditional channels (physical stores) is needed to understand the existence of an actual transition towards more sustainable fashion consumption patterns. Thus, the following hypothesis is tested:

#### H4

Consumers affected by the COVID-19 shock on traditional purchases are more likely to have the intention to purchase sustainable fashion products.

The unusual online purchasing behaviour is another factor that differentiates the COVID-19 from other previous crises (Laato et al., 2020). Online sales platforms started to work effectively before the COVID-19 outbreak (Safara, 2020) and the digitalisation is a complex phenomenon that was happening regardless of the pandemic (Silva & Bonetti, 2020). Nevertheless, the shock produced by the current crisis has affected purchasing behaviour not only in terms of reduced consumption but also in terms of different purchasing patterns (Laato et al., 2020), so that online consumer awareness and experience has increased (Gu et al., 2021). More than ever, we are now even more dependent on digital space and embedded in it for purchases (Hoelscher & Chatzidakis, 2021). Indeed, the first unavoidable effect of lockdowns in the fashion industry was the shift of purchasing from physical stores—many of which were forcibly closed—to online sales platforms (Amed et al., 2021).

The “online” revolution had a strong impact on the fashion industry consumption. Pang et al., (2021) showed that offline clothing consumption at mass markets decreased since the pandemic, whereas, at the same time, online clothing consumption increased. Niehoff (2022) argued that it is not clear if the COVID-19 pandemic has influenced online purchasing to create a more sustainable consumption pattern. Sadiq et al., (2021) highlighted that online consumers usually are different from consumers that purchase in physical stores. Therefore, there is still a lack of studies on the positive effect of COVID-19 on green purchase intention in online apparel purchasing channels. To verify that, the fifth hypothesis has been structured as follows:

#### H5

Consumers affected by the COVID-19 shock on online purchases are more likely to have the intention to purchase sustainable fashion products.

The pandemic may have strengthened the influence of the antecedents on sustainable purchase intentions due to the rise in knowledge and concern, as has already been observed in the tourism sector (Sobaih et al., 2021). However, existing studies have only investigated the direct relationship between COVID-19 and green purchases in physical stores (Orîndaru et al., 2021; Chae, 2021). Therefore, the positive mediating role of the COVID-19 pandemic on the antecedents of green purchase intention still needs to be investigated in traditional fashion purchasing channels. In this sense, we will test the following hypotheses:

#### H6a

COVID-19 shock on traditional purchases positively mediates the relationship between environmental knowledge and the intention to purchase sustainable fashion products.

#### H6b

COVID-19 shock on traditional purchases positively mediates the relationship between environmental concern and the intention to purchase sustainable fashion products.

#### H6c

COVID-19 shock on traditional purchases positively mediates the relationship between eco-friendly behaviour and the intention to purchase sustainable fashion products.

As previously stated, the post-pandemic growth in online purchasing is one of the most significant and clearest effects of the crisis (Amed et al., 2021). In fact, Gu et al., (2021) highlighted that COVID-19 fosters online shopping. Pang et al., (2021) showed an increase of online consumption of fashion products due to COVID-19. Moreover, Tran (2021) showed that pandemic fear positively moderates the relationship between economic benefits and sustainable consumption through e-commerce platforms. However, there is a lack of studies that investigate the positive mediating role of COVID-19 shock on online purchases, on the analysed antecedents of green purchase intention in the fashion industry. Consequently, we also tested the following hypotheses:

#### H7a

COVID-19 shock on online purchases positively mediates the relationship between environmental knowledge and the intention to purchase sustainable fashion products.

#### H7b

COVID-19 shock on online purchases positively mediates the relationship between environmental concern and the intention to purchase sustainable fashion products.

#### H7c

COVID-19 shock on online purchases positively mediates the relationship between eco-friendly behaviour and the intention to purchase sustainable fashion products.

### Conceptual model

**Fig. 1:**
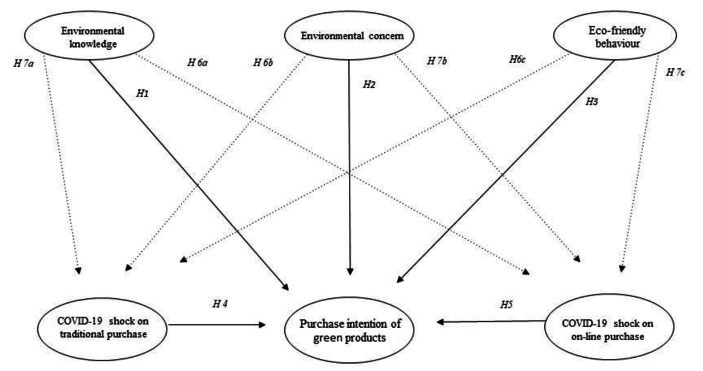
The Conceptual Model of the Study

Figure 1 illustrates the overall theoretical model with the hypotheses to be tested:

## Methods

### Data description

The data were collected from an online questionnaire of 1433 consumers in Italy from May 2020 to March 2021. The survey was structured similarly to other published works, and the sample size was consistent with prior empirical research (Sharma et al., 2020; Laato et al., 2020; Lee et al., 2014; Uddin and Kahn, 2018; Hartmann & Apaolaza-Ibáñez 2012).

To reduce potential response bias, we used various procedural remedies. First, the questionnaire was tested and validated by two experts on sustainability in the fashion industry. Second, anonymity was guaranteed for all respondents. The questions were checked to ensure they did not include any ambiguous or unfamiliar terms, vague concepts, and complicated syntax (Miles et al., 2014).

### Measurement

The constructs used items adapted from the literature measured on a five-point Likert scale ranging from 1 (“not important/agree”) to 5 (“the highest importance/agreement”) - see Appendix 1.

The environmental knowledge (EK) construct consisted of a set of three statements measuring the knowledge of environmental issues in the fashion sector adapted from Shen et al., (2012). The environmental concern (EC) construct was measured with a set of multi-items again adapted from Shen et al., (2012), with seven items measuring the environmental dimension. The eco-friendly behaviour (EFB) construct consisted of a set of three items adapted from Salem et al. (2020): purchase of products with recycled materials, reading of labels and reuse of containers. The scale for the green purchase intention (GPI) was made up of three items adapted from several authors (i.e., Nosi et al., 2014; Hustvedt & Dickson, 2009; Ajzen, 1991; Ajzen, 2015) measuring the intention to buy a sustainable product in the fashion sector. The measurement scales to test the direct effect of COVID-19 on traditional (COVonT) and online (COVonL) purchases consist of a set of multi-items used to assess the consumption reduction and paradigm shift in the fashion sector since the outbreak of the pandemic, previously tested among a group of experts. Specifically, COVonT measured by what percentage the health emergency related to the COVID-19 pandemic reduced shopping in physical stores and to what degree the outbreak changed people’s experience of buying fashion products in bricks and mortar fashion stores. COVonL is another variable, assessing by what percentage the outbreak led to an increase online in purchases of fashion products.

#### Control variables

Among the variables analysed in the literature, demographic characteristics such as age and gender are central (Rahman & Koszewska, 2020). Studies have shown that younger consumers are more inclined than older ones to make environmentally friendly purchases (Hill & Lee, 2012) and that women assume different consumption patterns than men in terms of green consumption (Uddin and Kahn, 2018; Dagher et al., 2015). To avoid interference, the model includes the use of control variables, namely gender, age, education, and income.

## Results

### Results of the measurement model

Table 1 reports the descriptive statistics and correlation matrix of variables tested in the study.

**Table 1:** Descriptive statistics, correlation and discriminant validity

		[1]	[2]	[3]	[4]	[5]	[6]	[7]	[8]	[9]	[10]
[1]	GPI	**0.831**									
[2]	ECFS	0.378	**0.774**								
[3]	EKAP	0.387	0.209	**0.861**							
[4]	EFB	0.505	0.386	0.512	**0.790**						
[5]	COVonL	0.185	0.192	0.183	0.162	**0.741**					
[6]	COVonT	0.205	0.181	0.202	0.164	0.498	**0.774**				
[7]	Age	-0.092	-0.080	0.039	0.000	-0.114	-0.043	**-**			
[8]	Education	-0.023	0.060	0.096	0.026	0.051	0.027	0.013	**-**		
[9]	Gender	-0.124	-0.114	-0.080	-0.072	-0.121	-0.045	0.088	-0.039	**-**	
[10]	Income	-0.102	-0.071	0.013	-0.049	-0.073	-0.040	0.290	0.098	0.111	-
	Mean	2.967	3.621	2.156	2.474	2.693	2.702	0.389	0.505	0.320	0.286
	St. Dev.	1.007	1.033	0.978	1.055	1.140	1.057	0.488	0.500	0.467	0.452
	Min	1	1	1	1	1	1	0	0	0	0
	Max	5	5	5	5	5	5	1	1	1	1

Note: N = 1,433. Correlation coefficients greater than 0.060 in absolute value are statistically significant at 95%. Correlation coefficients greater than 0.088 in absolute value are statistically significant at 99%. Values on diagonal are the square root of AVE.

Before testing the hypothesized model, internal reliability, discriminant validity, and convergent validity were verified in each construct (Table 2).

**Table 2:** Constructs and their measures

Constructs	Items	Std. Factor Loadings
Green Purchase Intention (GPI) α = 0.86 CR = 0.87 AVE = 0.69		
	GPI1	0.88
	GPI2	0.71
	GPI3	0.89
Environmental Knowledge (EK) α = 0.94 CR = 0.89 AVE = 0.74	EK1	0.82
	EK2	0.97
	EK3	0.78
Environmental concerns (EC) α = 0.91 CR = 0.91 AVE = 0.60		
	EC1	0.77
	EC2	0.82
	EC3	0.78
	EC4	0.86
	EC5	0.68
	EC6	0.69
	EC7	0.80
Eco-friendly behaviour (EFB) α = 0.82 CR = 0.83 AVE = 0.62		
	EFB1	0.82
	EFB2	0.85
	EFB3	0.69
COVID-19 shock on traditional purchase (COVonT) α = 0.83 CR = 0.83 AVE = 0.55	COVonT1	0.77
	COVonT2	0.83
	COVonT3	0.72
	COVonT4	0.63
COVID-19 shock on on-line purchase (COVonL) α = 0.88 CR = 0.88 AVE = 0.60		
	COVonL1	0.70
	COVonL2	0.91
	COVonL3	0.86
	COVonL4	0.69
	COVonL5	0.68

Cronbach’s α and the composite reliability (CR) examined the internal reliability. Cronbach’s α was higher than 0.7 in all constructs. Constructs assumed CR values above the usual threshold of 0.7 (between 0.83 and 0.9) (Bagozzi & Yi, 1998). These results confirm a high internal consistency of all the studied constructs. Appendix 2 shows the results of a factor analysis of all the items using a maximum likelihood estimation and promax rotation. Every standardized factor loadings exceeded the threshold of 0.5 (Chin, 1988).

The square root of the average variance extracted (AVE) (Fornell & Larcker, 1981) and cross-loadings verified a satisfactory discriminant validity: for each construct AVE square roots were higher than the correlation between the construct and each other (Fornell & Larcker, 1981) (Table 2). The factor loadings were thus higher than the cross-loadings.

The AVE values confirmed a satisfactory convergent validity because they were higher than 0.5 (Fornell & Larcker, 1981). These results show the acceptable reliability and validity of the measurements implemented in this study.

The mean variance inflation factor (VIF) was 1.30 (Appendix 3). This result shows the lack of multicollinearity among the variables as reported in the literature (Kutner et al., 2004).

### Results of the structural model

The study applied a structural equation model to test the hypotheses through STATA 15.1 software. The results of the path analysis on the hypothesized structural equation model are reported in Table 3. The model showed a good fit in line with all the threshold values recognized by the literature (Hair et al., 2009): CFI = 0.900; RMSEA = 0.066; SRMR: 0.048; *R*^*2*^ (*GPI*) 0.411.

**Table 3:** Structural model results

Paths	Model A		Model B	
	Std. Coeff.	S.E.	Std. Coeff.	S.E.
GPI				
← EK	0.160***	0.028	0.166***	0.028
← EC	0.129***	0.033	0.117***	0.033
← EFB	0.471***	0.040	0.477***	0.041
← COVonT	0.061**	0.029	0.057**	0.030
← COVonL	0.022	0.028	0.037	0.029
← Age	-0.118**	0.047		
← Education	-0.140**	0.044		
← Gender	-0.119**	0.048		
← Income	-0.087*	0.051		
COVonT				
← EK	0.162***	0.036	0.162***	0.036
← EC	0.187***	0.043	0.187***	0.043
← EFB	0.043	0.051	0.043	0.051
COVonL				
← EK	0.150***	0.035	0.150***	0.035
← EC	0.119**	0.041	0.119**	0.041
← EFB	0.079	0.050	0.079	0.050
*χ* ^*2*^	2554.681		2167.005	
*df*	356		260	
*p*	< 0.001		< 0.001	
RMSEA	0.066		0.072	
CFI	0.900		0.912	
SRMR	0.048		0.042	

* *p* < 0.100; ** *p* < 0.050; *** p < 0.001

The results of path analysis do not present any particular differences by including (Model A) or excluding the control variables (Model B) (Table 3).

H1, which identifies a positive relationship between environmental knowledge and intention to purchase sustainable fashion products was supported because EK is related to GPI. The models also showed a statistically significant relationship between EC and GPI. Therefore, consumers with a high environmental concern are more likely to be willing to purchase sustainable fashion products, thus verifying H2. The results also showed that the important relationship between EFB and GPI is significant. This means the implementation of eco-friendly behaviour triggers the intention to purchase sustainable fashion products.

The models showed that the relationship between COVonT and GPI is significant (H4), whereas the relationship between COVonL and GPI is not significant (H5). Therefore, the COVID-19 shock appears to have impacted on traditional purchases by slightly increasing the intention to purchase sustainable fashion products.

The models proved that COVID-19 shock on traditional purchases mediates positively the relationship between two of the three antecedents of purchase intention for sustainable fashion products (i.e., environmental knowledge and environmental concern), and purchase intention. Consequently, H6a and H6b were supported.

This mediation was not confirmed for the relationship between eco-friendly behaviour and purchase intention (H6c). The same results can be identified for H7a and H7b, which verified the positive mediation of COVID-19 shock on the relationship between environmental knowledge and environmental concern, and purchase intention for sustainable fashion products online, whereas the mediation between relationship between eco-friendly behaviour and purchase intention is not significant (H7c).

**Fig. 2:**
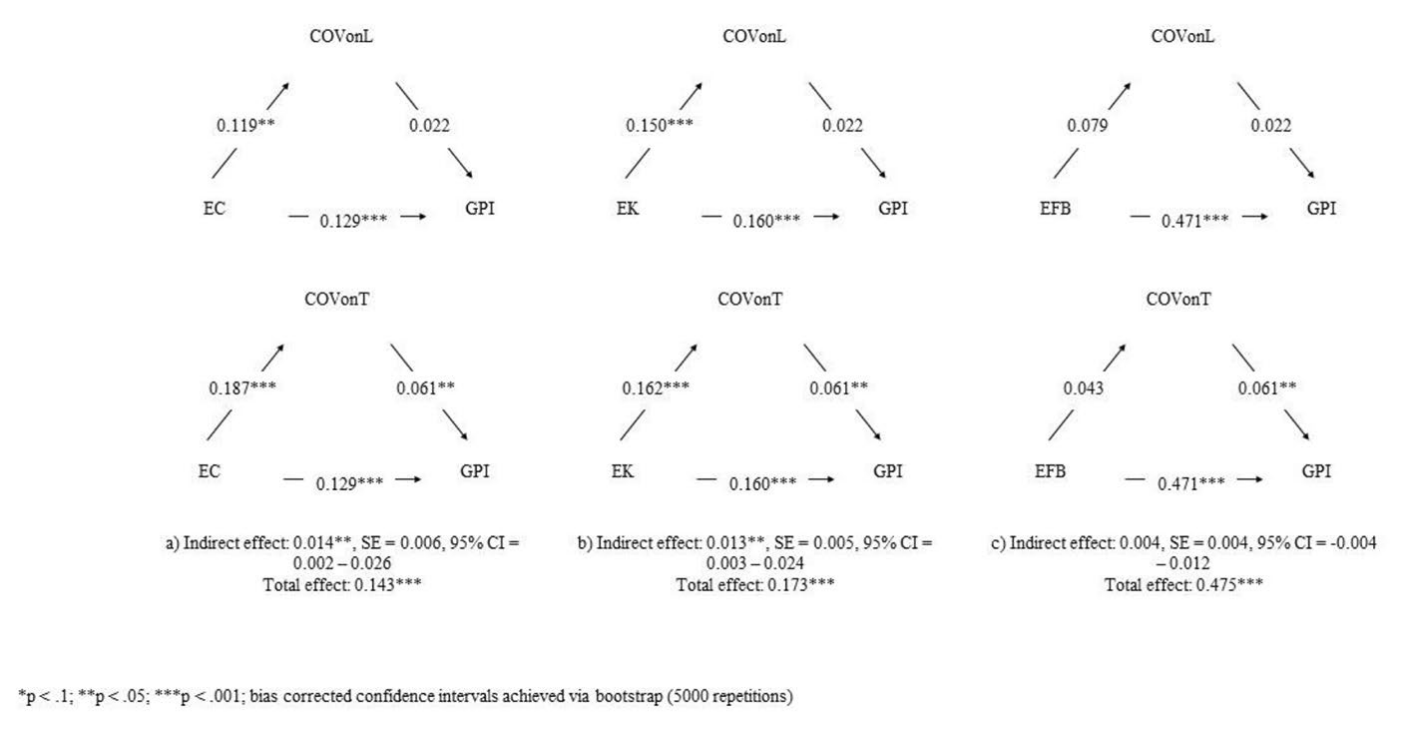
Effects Decomposition for indirect paths

Preacher et al., (2006) suggested that the measurement of the decomposition of indirect paths. Figure 2 provides 95% bootstrap confidence intervals for these indirect effects by showing partial mediation between variables (Little et al., 2007). In fact, both indirect and direct relationships between two out of three antecedents, i.e., EC and EK, and GPI are both significant.

EC and EK have a direct effect on the GPI but also a mediated effect because the COVID-19 shock fostered the purchase of green fashion products.

Our model considers two mediators, distinguishing between the effect on the online channel and the effect on the traditional channel.

The size of the indirect effect of the three antecedents is therefore the result of two mediation paths: one traditional and the other online.

In the case of EC, the indirect effect represents about 10% of the total effect: about 3% online and 7% traditional.

In the case of EK, the indirect effect represents about 7.5% of the total effect: about 3% online and about 4.5% traditional.

These results show the dimension of how much the COVID-19 outbreak may have accelerated the propension to buy sustainable fashion products.

## Discussion

The study highlights that the COVID-19 shock mediates positively the relationship between environmental knowledge and green purchase intention regardless of the type of selling channel (traditional or online), by confirming the positive effect of mediation factors that strengthen the capacity of environmental knowledge to foster green purchase intention.

These results provide further support to previous studies that found a mediation between knowledge and green purchase decision making through attitudes toward green products (Indriani et al., 2019). Our paper highlights the boosting effect of a shock on existing environmental knowledge resulting from a long-term stratification process for understanding the ecosystem dynamics and the environmental impact of production and consumption.

The positive mediation effect of the COVID-19 shock on environmental concern and green purchase intention confirm that the potential limits of an individual’s affective assessment of environmental issues can be partially overcome by an exogenous shock that highlights and then strengthens the casual relationship between environmental issues and purchase choices (Newton et al., 2015).

The lack of the significance of the mediation effect of COVID-19 on eco-friendly behaviour and green purchase intention highlights that the implementation of eco-friendly behaviour determines an influence on green purchases that can combat the effects of external events or shocks. This result confirms the direct effect on green purchase intention by highlighting consumers’ education role played by eco-friendly behaviour (Abdul-Muhmin, 2007).

By identifying the positive effect of environmental knowledge, environmental concern and eco-friendly behaviour on green purchase intention, this study highlights that the decision-making process for purchasing sustainable fashion products needs time to stratify specific knowledge and beliefs related to the environmental impact of purchase choices (Gam, 2011). Therefore, past experiences, knowledge and beliefs related to sustainable consumption and production represent a starting point for designing ad hoc strategies and actions for increasing green purchases.

Finally, the study shows that the COVID-19 shock on traditional purchase channels exerts a significant positive effect on green purchase intention results. Therefore, by changing the cognitive approach of the purchase channel, the COVID-19 shock influences not only the frequency of visits but also consumers’ attitude in physical stores (Brydges et al., 2020). This highlights the importance of implementing green purchases because consumers have increased their awareness of the effect of their purchases on the environment and the society. On the other hand, the relationship between the COVID-19 shock on online purchases and green purchase intention is not significant because digitalisation is a complex phenomenon (Silva & Bonetti, 2020) and might be controversial in terms of environmental impacts such as the carbon dioxide emissions generated by express deliveries (Xie et al., 2020).

These findings show that COVID-19 affected purchase decisions but did not necessarily foster green purchases in the fashion industry. The COVID-19 shock was a crucial event in the stratification process of knowledge and beliefs in the green purchase decision process. In order to tackle future exogenous shocks such as the COVID-19 outbreak, fashion companies should strengthen their capability to convey their efforts they are making to reduce the environmental impacts of their products.

This study contributes to extending the current understanding of green purchase decisions by analysing the impact of an exogenous and complex shocks, i.e., the COVID-19 and the role of three antecedents of green purchase intention, i.e., environmental knowledge, environmental concern, and eco-friendly behaviour.

First, the study provides evidence of the direct and indirect impact of the COVID-19 shock on green purchases by enlarging the knowledge of previous studies that analysed the relationship between economic crisis and green consumption (Ozdamar Ertekin et al., 2020; Tilikidou & Delistavrou, 2014). Indeed, the COVID-19 outbreak represents a complex and important shock that has affected several economic sectors (Donthu & Gustafsson, 2020), challenged individuals’ routines, behaviours, and social relations (Van Bavel et al., 2020), and altered the operation of organisations (Rai et al., 2020).

Second, this analysis sheds light on the effect of the COVID-19 shock on traditional and online purchase channels that might differentially affect consumers’ green purchase choices. Therefore, the study contributes to the existing debate about how different purchase channels such as physical stores and online shops play a pivotal role in the decision process for purchasing sustainable fashion products. This study emphasizes the need for a seamless integration of offline and online retail to communicate fashion companies’ efforts to reduce their negative environmental impacts.

Third, this study tested the direct effect of environmental knowledge, environmental concern, and eco-friendly behaviour, but also their mediated role (Groening et al., 2018), on green purchase intention. The results showed how constructs resulting from long-term stratification processes and individuals’ actual contribution to the conservation of the environment interact directly and indirectly with green purchase intention. In particular, the findings recognize the reinforcing role played by mediating factors in terms of environmental knowledge and environmental concern, whereas eco-friendly behaviour is able to strongly affect green purchase intention without any mediation.

## Conclusion

The study investigated the direct and indirect effect of the COVID-19 on green purchase intention thereby contributing to the literature on sustainable consumption but with useful managerial implications.

The significant role played by the COVID-19 outbreak in fostering green purchase intention highlights two implications for companies offering and promoting sustainable fashion products.

First, companies that have already implemented actions for reducing the negative environmental impacts of their processes and products were potentially favoured by the COVID-19 outbreak in terms of selling sustainable fashion products. The exogenous shock of COVID-19 did not have a detrimental impact on companies that have traditionally invested in environmental sustainability and demonstrated their commitment to the environment, and they even managed to strengthen their market segment of sustainable fashion products. Therefore, companies’ strong and long experience in sustainable fashion production should be communicated and promoted effectively because it represents an effective assurance against complex exogenous shocks affecting their markets. Consumers also have all the necessary information for carrying out an effective and informed purchasing decision process.

Second, fashion companies aiming to start the shift to sustainable production should set up a long-term strategy for the implementation and communication of actions for reducing the negative environmental impact of their processes and products. By assuming this perspective, companies will be able to signal their consolidated commitment to environmental sustainability to consumers even in the case of exogenous events and shocks, avoiding the need for greenwashing. The ability to implement long-term promotion and communication will thus be rewarded by consumers with high environmental concern and environmental knowledge because these antecedents result from long-term stratification processes.

The results should be interpreted considering certain limitations. First, the study adopted a cross-sectional perspective. Future studies should consider gathering longitudinal data, which can offer valuable insights into the dynamics of relationships among the study constructs and particularly into the long-term impact of COVID-19 on the green purchase intention. In addition, this study focused on a specific country. Since environmental issues are not confined to specific nations and sectors, a replication of this research in other countries, with different economic, socio-cultural, and political–legal conditions, and sectors would test its external validity.

Second, the study used purchase intentions instead of green purchase behaviour. Even though intention is an important construct to contribute to the analysis of decision-making processes, a gap often exists between consumers’ intentions and their actual behaviours (Sheeran, 2002). Therefore, future studies could use actual purchase data to support our findings.

## Electronic supplementary material

Below is the link to the electronic supplementary material.

Supplementary Material 1
