# Supplementary material for: The mediating role of exogenous shocks in green purchase intention: evidence from italian fashion industry in the Covid-19 era
Source: Ital. J. Mark. 2023 Jan 19;2023(1):59–79. doi: 10.1007/s43039-023-00065-4 (PMC9850337; doi:10.1007/s43039-023-00065-4)
Supplement: Supplementary file 1 — Supplementary Material 1 [file 43039_2023_65_MOESM1_ESM.docx]

**Appendix 1:**

| Constructs | Item Description | Sources |
| --- | --- | --- |
| Green Purchase Intention (GPI) |  |  |
|  | GPI1: I intend to buy a sustainable fashion product next time I go fashion shopping | Nosi et al., 2014; Hustvedt and Dickson, 2009; Ajzen, 1991; Ajzen, 2015 |
|  | GPI2: I will make an effort to buy a sustainable fashion product next time I go fashion shopping |  |
|  | GPI3: I want to buy a sustainable fashion product next time I go fashion shopping |  |
|  |  |  |
| Environmental Knowledge (EK) |  |  |
|  | EK1: I believe I am informed about environmental issues in the fashion industry | Shen et al., 2012 |
|  | EK2: I know sustainable fashion well |  |
|  | EK3: I know the retailers who sell sustainable fashion |  |
|  |  |  |
| Environmental concerns (EC) |  |  |
|  | EC1: Products made with organic / biological / natural fibers, use of vegetable dyes, etc. | Shen et al., 2012. |
|  | EC2: Products made using recycled materials |  |
|  | EC3: Products made with biodegradable materials |  |
|  | ECS4: Products made by companies that recycle raw materials and minimize energy and water waste |  |
|  | EC5: Products made without exploiting the fur of animals, their skin or hide |  |
|  | EC6: The dyes and bleaches used to make the fashion products have not been tested on animals |  |
|  | EC7: The by-products of garment making (e.g. waste water) have been managed in a way that does not pose a threat to wildlife (e.g. fish, birds, etc.) |  |
|  |  |  |
| Eco-friendly behaviour (EFB) |  |  |
|  | EFB1: I generally buy products made with recycled materials | Salem et al., 2020 |
|  | EFB2: I generally read product labels to check if they respect the environment |  |
|  | EFB3: I generally reuse empty product containers / packages |  |
|  |  |  |
| COVID-19 shock on traditional purchase (COVonT) |  |  |
|  | COVonT1: Reduced the ability to try / wear products | Verma and Gustafsson, 2020; Hasbullah et al., 2020; Ozdamar Ertekin et al., 2020; Sheth, 2020; Pantano et al., 2020; Brzustewicz and Singh, 2021; Orîndaru et al., 2021; Chae, 2021; Brydges et al., 2020. |
|  | COVonT2: Reduced the ability to directly evaluate product materials |  |
|  | COVonT3: Reduced the chance of being recommended by the store's salespeople |  |
|  | COVonT4: Reduced the possibility of being accompanied by family or friends during the purchase process |  |
|  |  |  |
| COVID-19 shock on on-line purchase (COVonL) |  |  |
|  | COVonL1: Necessity due to lockdown | Pang et al., 2021; Niehoff, 2022; Sadiq et al., 2021; Amed et al., 2021; Laato et al., 2020 ; Gu et al., 2021; Hoelscher and Chatzidakis, 2021. |
|  | COVonL2: Fear of infection (linked to lack of social distancing) |  |
|  | COVonL3: Fear of infection (linked to mistrust of product sanitation) |  |
|  | COVonL4: Increase in the time required to purchase in the store (queue at the entrance, etc ...) |  |
|  | COVonL5: Reduction of the pleasure associated with the in-store shopping experience |  |

**Appendix 2:**

| Rotate Factor Loadings (promax rotation) | | | | | | |
| --- | --- | --- | --- | --- | --- | --- |
|  | Factor 1 | Factor 2 | Factor 3 | Factor 4 | Factor 5 | Factor 6 |
| GPI1 | 0.792 |  |  |  |  |  |
| GPI2 | 0.654 |  |  |  |  |  |
| GPI3 | 0.820 |  |  |  |  |  |
|  |  |  |  |  |  |  |
| EC1 |  | 0.734 |  |  |  |  |
| EC2 |  | 0.782 |  |  |  |  |
| EC3 |  | 0.729 |  |  |  |  |
| EC4 |  | 0.813 |  |  |  |  |
| EC5 |  | 0.720 |  |  |  |  |
| EC6 |  | 0.754 |  |  |  |  |
| EC7 |  | 0.837 |  |  |  |  |
|  |  |  |  |  |  |  |
| EK1 |  |  | 0.795 |  |  |  |
| EK2 |  |  | 0.919 |  |  |  |
| EK3 |  |  | 0.762 |  |  |  |
|  |  |  |  |  |  |  |
| EFB1 |  |  |  | 0.659 |  |  |
| EFB2 |  |  |  | 0.641 |  |  |
| EFB3 |  |  |  | 0.588 |  |  |
|  |  |  |  |  |  |  |
| COVonL1 |  |  |  |  | 0.721 |  |
| COVonL2 |  |  |  |  | 0.881 |  |
| COVonL3 |  |  |  |  | 0.843 |  |
| COVonL4 |  |  |  |  | 0.689 |  |
| COVonL5 |  |  |  |  | 0.677 |  |
|  |  |  |  |  |  |  |
| COVonT1 |  |  |  |  |  | 0.681 |
| COVonT2 |  |  |  |  |  | 0.743 |
| COVonT3 |  |  |  |  |  | 0.756 |
| COVonT4 |  |  |  |  |  | 0.649 |

| **Appendix 3:**  *VIF scores and tolerance* (all variables and only principal covariates) | | | | |
| --- | --- | --- | --- | --- |
| **Variables** | **VIF scores** |  | **VIF scores** |  |
| GPI | 1.51 |  | 1.48 |  |
| EC | 1.28 |  | 1.26 |  |
| EK | 1.45 |  | 1.42 |  |
| EFB | 1.69 |  | 1.69 |  |
| COVonL | 1.39 |  | 1.36 |  |
| COVonT | 1.37 |  | 1.37 |  |
| Age | 1.12 |  |  |  |
| Education | 1.03 |  |  |  |
| Gender | 1.04 |  |  |  |
| Income | 1.12 |  |  |  |
| Mean VIF: 1.30. Condition number: 16.091 | |  | Mean VIF: 1.43. Condition number: 13.620 | |
